# Recent Advances in Surgery
*A Paper read before the Society on Wednesday, 8th February, 1939.


**Published:** 1939

**Authors:** W. H. Ogilvie

**Affiliations:** Honorary Surgeon, Guy's Hospital and Royal Masonic Hospital


### RECENT ADVANCES IN SURGERY.*

BY

W. H. Ogilvie, M.D., F.R.C.S.,

*Honorary Surgeon, Guy's Hospital and Royal Masonic Hospital*

We often speak somewhat glibly of recent advances. But "recent" may mean anything from the last five years to the last fortnight, and the adjective is almost a direct negative of the noun it qualifies. When a thing is recent it is impossible to say whether or no it is an advance; when its value has really been established, it is no longer recent. For by an advance I must insist that we mean something better and not merely something newer, and it is indeed difficult to distinguish the good from the merely new. It is not enough to read the communications in the journals; we must also know something of the ability, experience and integrity of the writer. For to-day the methods of journalism and high pressure business have invaded even the world of science. The young surgeon who would make a reputation need no longer set out to build it up by research and honest careful work. He gets a secretary to run through American and foreign literature, a professional illustrator working constantly on drawings, photography, slides, and films, and an impecunious registrar to do the writing. As the nominal head of this syndicate he packs the journals with his articles, the publishers' shelves with his volumes, and the students' heads with ill-digested statements. What stands declared as an advance may therefore be no more than the untried assumption of some second-rate theorist in another continent.

* A Paper read before the Society on Wednesday, 8th February, 1939.

Because it is in many ways the least important aspect of surgery, I would first say something of surgical handicraft. Operating has become incredibly better within the last fifteen years, and the young men who are serving their apprenticeship to-day, if they continue to work hard and to keep humble, should all become the equals, in technical achievement at any rate, of the greatest masters of the past. Part of this improvement is due to advances in architecture and engineering : the tables, the lights and the instruments with which the modern surgeon works are all about as perfect as they can be. Part of it is due to increasing standardization in operative procedures. There are still any number of different ways, all permissible, in which an operation can be done. It is remarkable in such a set piece as a sub-total thyroidectomy, where the object of attack and its surroundings are absolutely constant in their mutual relationships in every patient, where the same four arteries and six veins are always encountered and the same two nerves always avoided, how in different centres surgeons of equal skill use different methods. But pretty well all the ways are known, all have been tried and described, and the really bad ones have all been discarded ; the young surgeon now loses no time in experimenting, but has merely to select the method that appears best to him. Most of all, the improvement is due to greater opportunities offered at an earlier age. Posts are no longer won by waiting and teaching alone, but students of promise are trained to operate by apprenticeship while their brains are receptive and their fingers nimble, and they learn to use their hands with that deft certainty and artistry that can never be acquired by those who start on any craft in middle age.

Asepsis is interpreted far more rigorously to-day than even a few years ago. It is more than sixty years since Lister preached his gospel, and from the beginning of the present century all operations have been conducted under avowedly sterile conditions. But only lately has the difference been appreciated between healing by "first intention" and hundred-per-cent., copper-bottomed, first intention healing. In the first the wound never suppurates and the scar is covered by epithelium when the stitches are removed; but the patient runs a slight temperature for a few days and dislikes moving the injured part, the site of the incision is a little raised and a little warmer than its surroundings, the scar is two or three millimetres in diameter and reddish purple. In the second there is no constitutional disturbance, pain is unusual, the parts look normal right up to the incision, and the scar is a hair line by the fourth day, and practically invisible by the fourth week. Ten years ago such healing was the exception; it is now the rule.

In asepsis, preparation of the skin is unimportant and largely a matter of fashion, yet it is a ritual that marks the individuality of the clinic. Speaking surgically, the continental abdomen is white, the British abdomen brown or blue, the American one pink or red; in other words the French and Germans pin their faith to spirit, the English to iodine or the aniline dyes, the Americans to mercury preparations.

The two dangers at an operation in a properly run theatre on a patient from a properly run ward (that is, where preparation and sterilization can be trusted) are the entry of air-borne organisms, and droplet infection from a spectator or member of the team. Those who fear the first insist on filtered air in the theatres; others believe that such organisms are scanty and of low pathogenicity, and that they can be excluded or at any rate rendered innocuous by ordinary precautions, and particularly by careful handling of the tissues. If nothing is left at the end of an operation but clean-cut surfaces carefully approximated, and there is no devitalized tissue or blood on which they can grow and no dead space where such culture medium can accumulate, any bacteria that may have entered by chance will be killed before they can multiply. Among this school there is a definite tendency to use very fine thread or silk in place of catgut for ligatures and for the majority of sutures. Catgut is bulky, unreliable, irritating and sterilized with difficulty. It has one good quality only, that it is eventually absorbed, but it disappears with a scuffle of leucocytes in the midst of an œdema that can be felt through the surface. Thread of 100 gauge will do the work of No. 1 catgut ; and since knots are secure and ligatures may be cut short, it only takes up one-hundredth of the bulk. It is inert and needs no chemicals, but remains unresented among the tissues. Cranial surgeons have shown repeatedly that a wound closed with interrupted stitches of fine silk can be reopened at any time and will present the appearance of fresh undisturbed tissues. Thyroidectomies done with the same material show an equal improvement over a series done with catgut.

Droplet infection, however, is a very real danger. The human throat always contains streptococci, and may harbour hæmolytic strains of extreme virulence even when the carrier is apparently in perfect health. A few such organisms introduced into an operation field may set up a peritonitis or meningitis that is fatal within forty-eight hours. Such infection can be avoided entirely by the provision of proper masks ; these may be made impervious to air and moisture by a layer of cellophane, so that the expired air is deflected backwards past the ears; or they may be of such a thickness of gauze, ten layers having been found a minimum for this purpose, that no moisture can penetrate them. In any case the mask must cover the nose, and every unsterile person who approaches the patient, particularly the anæsthetist, must wear one.

While the members of a surgical team can be trained in every precaution against infection, the same cannot apply to spectators, who may be untrained students or casual visitors, and who can contaminate the operation field at a considerable distance, particularly if they are accommodated in stands or galleries above the level of the theatre floor and if they shout their questions. The tendency in modern theatre blocks is to segregate spectators entirely. The days are gone when an operation was a *tour de force* performed on rare occasions, when crowds assembled to see a leg come off or an abdomen opened, when the word "theatre" seemed the apt description for a room designed to allow the maximum number to view the spectacle. Operations are performed every day in every theatre of a hospital. They are mostly standardized procedures that are described with great detail in text-books of operative surgery, and the visitor gains little by watching unless he is near enough to see the details of the surgeon's technique. Such positions are reserved for members of the team in training, and one or two expert and privileged friends of the surgeon. Medical students, post-graduates, and visiting societies must be placed in galleries; and in all new theatre blocks, such as that being built at the Massachusetts General Hospital, these galleries are part of a spectators' unit, on the floor above and entirely separate from the operating unit. On the surgical floor, which is limited to surgically clean personnel and materials, are the theatres on one side of the passage, changing and store-rooms on the other. On the visitors' floor are registration and cloak rooms, rest room, and educational demonstrations above the changing rooms, while on the opposite side are a series of galleries, over the door of each of which a signal lights up to show when an operation is in progress. This gallery has a plate glass wall over the theatre through which, with field glasses, the image of the operative field can be brought to within two feet of the eye : but it is entirely sealed off, so that spectators can wear ordinary clothes, and can gossip or smoke without disturbing the surgeon. Communication is by two-way microphones and loud speakers, so that instructions from the theatre and questions from the gallery are relayed in that nasal bleat that seems inseparable from mechanical voice reproduction. A disadvantage is that occasionally a chance aside may be bellowed fortissimo, to the confusion of the speaker.

Among the dangers increasing the risk of operation, hæmorrhage stands out as the most dramatic, and also the most easily combated if we have the blood. The dangers of transfusion are known, its technique mastered. But because the supply of donors is limited, and confined almost entirely to voluntary helpers upon whose humanity we do not wish to impose unduly, we are apt to limit the use of transfusion to those cases where we feel there is actual danger unless it is done, and hesitate to employ it in the many where it might make operation much safer, or aid recovery considerably. As so often happens the remedy is simple and ready to hand. At each birth, an average of 125 c.cm. of blood is at present thrown away in the placenta. This can be expressed, grouped, citrated and kept in the refrigerator, where it will remain in excellent condition for at least eight weeks. This placental blood is not only healthy and free from extraneous proteins, but it contains one and a half times the normal proportion of red cells. Further, since the distribution of blood groups in the new-born is the same as that of the general population, this free blood reserve from the labour ward will be qualitatively adjusted to the demand.

Placental blood has been used successfully at Montreal for several years : and though British workers writing within the last few weeks have stated that the amount to be obtained is not sufficient to justify the trouble involved, and that infection is not easily avoided, the method has so many advantages that it should not be abandoned lightly. The preservation of blood is likely to assume increasing importance in the future. During the war, reserves were collected at some of our Casualty Clearing Stations before a big engagement, and kept successfully for a few days. But the technique of storage has since been elaborated by the Moscow Institute of Hæmatology, where it is claimed that corpse blood may be preserved for at least two months by mixing it in equal quantities with a fluid that is roughly Ringer's solution with sodium citrate added. The use of corpse blood has a ghoulish sound to our ears, and it is unlikely that it could be openly advocated or employed in any western country. But the technique is equally applicable to freshly drawn blood from volunteers. The medical services on the Republican side in Spain organized a system whereby healthy men on leave or in factories gave their quota to a collecting centre, whence supplies were dispatched in large containers to the advanced ambulance units. Should an emergency ever come in this country, transfusion would immediately become one of the most important measures to save life among the casualties of an air raid, in circumstances where the leisurely collection of blood from individual donors would be impossible.

Perhaps as the outcome of this gradual but very definite improvement in surgical handicraft, there has come about a more obvious appreciation of the fact that surgery is a highly skilled art. The public have always been ready to grade athletes and musicians, to pay two guineas to hear Kreisler play the "Humoresque" while they consider the street violinist well rewarded with twopence for rendering the same piece; but they have valued all surgeons alike whether they possess a pass degree or a high diploma, whether they are newly qualified or have worked for many years on the staff of a hospital. Even our own profession has been wont to judge results as those of the operation rather than the operator, and to look on the man with a mortality of under 5 per cent. for gastric surgery as a good liar rather than a good surgeon. But we find now in many quarters an acknowledgement of the fact that surgery is not just surgery, but excellent, good, bad, indifferent or deplorable; particularly is this accepted in the surgery of cancer.

A fair comparison between the alternative methods of treatment in this disease has always been difficult because of the difficulty of finding reliable and impartial statistics. Every surgeon knows that good radium treatment will give better results than bad surgery, and that good surgery is preferable to bad radium. But official publications from radium centres have been vitiated by two things; that the cases are graded, on grounds which are not apparent, into an "operable" group which is far smaller than the proportion operated upon in a surgical clinic and which contains all the good results, and "inoperable" and "advanced" groups for the failures; and that the results are compared with those of very indifferent surgery. I was, therefore, interested, on visiting the Christie Institute at Manchester last November, to find that Ralston Paterson considers that, as far as the glandular field in the neck is concerned, the safety conferred by a block dissection well performed is greatly in advance of what any form of radiation can offer. The emphasis is on "well performed," and this, at the Christie Institute, means performed by Mr. Douglas. I had the good fortune to see this surgeon do one such dissection, and I should agree that it was a complete and anatomical clearance of all the lymphatic structures on that side of the neck more skilful and more thorough than anything I had seen in this country or the Continent; and could well understand the aphorism I had heard that afternoon, that recurrence after a block dissection indicated that the operation had not been done by Douglas. In the case of the breast, I had always been dissatisfied by Keyne's comparison of the five-year cure-rate for operable cases treated by radium, one of 63 per cent., with a similar figure for early cases treated by the radical operation: his figures for surgery were taken from the massed results of a large hospital. It is therefore very satisfactory indeed to see Gordon Taylor's carefully recorded personal figures, giving a ten-year cure-rate of 85 per cent. in cases without glands; a remarkable tribute to his well known technical skill and courage, and a stimulus to all those whose results are less satisfactory to study his methods. This is not a unique achievement, for a similar five-year cure-rate of 85 per cent. was reported ten years ago from the Leeds Infirmary.

While most surgical advances are in method, new problems arise from time to time. A group of diseases that was unknown ten years ago, but that is of considerable interest at the present time, is that of the functioning adenomas of the ductless glands ; tumours that give symptoms, not only from their presence and from increased pressure, but from producing some hormonic substance in excess. At the end of the war the only example of this group that was known was the "pituitary tumour" associated with acromegaly and gigantism. We now know that this particular syndrome is associated with an adenoma of the acidophil cells of the anterior lobe, that adenomas of at least two other kinds of cell may be found in the pituitary, and that similar functioning adenomas occur in other ductless glands, giving strange clinical pictures and sometimes affording the pathological explanation of a disease long known.

The chief members of this group, and their leading symptoms, are :—

| ADENOMA. | SYNDROME. |
|---|---|
| *Pineal* .. .. .. | Dwarfism with sex precocity. |
| *Pituitary : Acidophil* .. | Gigantism when occurring during the growth period ; acromegaly when appearing in adult life. |
| *Pituitary : Basophil* .. | Sex dystrophy ; amenorrhoea in the female, impotence in the male, excess of hair in both. |
| *Pituitary : Neutrophil* | Pressure symptoms only : endocrine picture of pituitary deficiency. |
| *Parathyroid* .. .. | Mobilization of calcium : disappearance from skeleton, increase in blood and urine, increased phosphatase, decreased phosphorus. |
| *Adrenal : Cortex* .. | Precocious sex and muscular development in males. Virilism in females, with amenorrhœa, change of voice, and male distribution of hair. |
| *Adrenal : Medulla* .. | Paroxysmal hypertension. |
| *Pancreas : Islet* .. | Hyperinsulinism and insulin fits. |

These tumours are mostly rare, and I do not wish to take up your time with a discussion of rarities, but I will touch on four of them that present points of particular interest.

The clinical picture of the parathyroid adenomas has been made familiar to all of us by the stimulating series of papers coming from the pen of Donald Hunter at the London Hospital, and numbers of cases are now reported every year. The steps by which this picture was put together are, however, less familiar, and are worth recording. Von Recklinghausen in 1891 described the condition of general fibrosis of the skeleton, with multiple cyst formation, which is known by his name, and which has been reported from time to time as a rare curiosity. In 1904 Askanazy reported a case of von Recklinghausen's disease associated with a parathyroid tumour. At the time this observation passed unnoticed, but as more cases were reported, it became recognized that there was some association between the two conditions. It was thought, however, that the parathyroid enlargement was secondary to the bone disease—an attempt to lay down more calcium. Acting on this theory Mandl of Vienna in 1926 inserted multiple parathyroid grafts into a patient with this disease, and found to his surprise that the condition got worse instead of better. He then exposed the thyroid, and found a small adenoma of the parathyroid, which he removed. Almost immediately calcium began to be laid down in the softened bones and the cysts to be filled up. As a result of this historic operation, we now recognize the clinical picture of a parathyroid tumour. There is wide-spread absorption of calcium in all the bones of the skeleton: in addition, multiple foci of osteitis fibrosa, with cysts or giant cell tumours, are found in the long bones and the skull:

the calcium content of the serum is raised and the phosphorus lowered; in the urine there is a greatly increased output of both calcium and phosphorus.

When this picture is encountered, a parathyroid tumour can be diagnosed with certainty, removal of which is followed immediately by a return to normal of the calcium in the blood and urine, and later by deposition of calcium throughout the skeleton. But Oliver Cope, of the Massachusetts General Hospital, has demonstrated two other clinical manifestations of parathyroid over-activity. The first may be called the "poor negative" syndrome. Calcium is removed from the skeleton, but without any bending or localized cyst formation. The patient may be admitted to hospital suffering from a fracture caused by ordinary violence and is sent for X-ray. The films when returned show a very poor shadow of the bones; indeed, if they are not accompanied by an apology from the radiologist, they usually evoke a bantering request from the surgeon for something a bit better next time. When successive films turn out to be equally poor, it is realized that the fault is in the bones rather than the apparatus, and an investigation of the calcium metabolism will settle the diagnosis. The second atypical expression of hyperparathyroidism is the formation of multiple renal calculi. These less common indications of a parathyroid tumour must be borne in mind, since the most important aspect of their treatment is the removal of the tumour; if this is not done, other fractures will occur, and fresh renal calculi will form after the first have been removed.

The Islet tumours, or $\beta$-cell adenomas of the pancreas, are important because they are commoner than we realize, and because their victims, who might be cured if the diagnosis were known, are often going about with the false label of "epilepsy" or *petit mal*, or are regarded by their fellows as "just peculiar." The typical history is that an individual, previously normal, begins to suffer from "fits" or fainting attacks. These attacks are apt to come in the morning, after severe exertion, or when for any reason the patient has remained unusually long without food. It is characteristic that the sufferer recognizes that he can avert a threatened attack by taking food, and in many cases realizes that a sweet drink is even more effective than a meal. The attack is preceded by a feeling of something amiss. It can be delayed for some time by will power, and the patient can usually finish what he is doing or seek help. At the beginning of an attack the conduct may be irrational. Unconsciousness supervenes, during which there may be twitchings of the limbs or attempted movements may be done in a violent manner, but there are no true convulsions. The patient starts to revive as soon as the sweet drink is given, and will often finish the drink himself. With the advent of insulin treatment, the similarity to the effects of an overdose of insulin at once suggested the correct pathology, and the syndrome was first recognized by a doctor sufferer, who died at the Mayo Clinic in 1927. The diagnosis is confirmed by an estimation of the blood-sugar, which shows a fasting value of 25 to 60 mgm. per 100 c.cm. instead of the normal 100 mgm., and of the sugar tolerance, in which a return to a subnormal blood figure occurs in four or five hours.

The treatment is by operation, with exposure of the pancreas and removal of the tumour. Three such operations were successfully performed by Howland, Fisher, and Graham, in 1929, 1930, and 1931, and in each case the blood-sugar returned to normal within twenty-four hours. But several surgeons failed to find any tumour in typical cases: and Graham, after his first success, was disheartened by drawing a blank in his second. The subsequent history of this case is an interesting example of how progress in one branch of surgery may lead to advance in another. Graham, whose work at Toronto on the surgery of the stomach is well known, became converted to Finney's operation for lesions at the pylorus, and was interested to find how well the whole duodenum could be mobilized by proper dissection; and further, how, when the duodenum was free, the head of the pancreas came up with it and could be palpated between the fingers. He sent for his former patient with hypoglycæmia, re-opened the abdomen, and was delighted to find in the head of the pancreas the tumour which he had been unable to feel at the previous laparotomy. It is now believed that these $\beta$-cell tumours show a preference for the head of the pancreas, and that one will always be found in a case giving the typical history of hypoglycæmic attacks.

The functioning tumours of the adrenal medulla are much rarer than the adenomas of the parathyroid and the pancreas. Three only have been encountered at the Mayo Clinic. They give rise to attacks of arterial hypertension, that are accompanied by symptoms very like those of paroxysmal tachycardia—headache, dizziness and a sensation of bursting in the chest. The attacks are brought on by exertion or excitement, and are characterized by a sudden rise of the systolic blood pressure by as much as 100 mm., and an equally sudden drop. The diagnosis is confirmed by laparotomy, and the attacks are cured by removal of the tumour.

Tumours of the adrenal cortex have long been recognized as associated with sex precocity and exaggerated muscular development in children, and the older generation will remember the striking picture of such an infant in Bland Sutton's *Tumours Innocent and Malignant.* To-day their relation to virilism in women is the subject of intensive study in all parts of the world, and particularly in London, where my friend Broster has made this curious subject his special study. Bearded women have been known throughout the ages. Sometimes they were canonized as saints, sometimes they were burnt at the stake ; in our day they are objects of pity, for they are rendered self-conscious by their misfortune, and excluded from many of the joys of social intercourse. Cortical tumours have been found in many, and their removal has restored the patients to normality. Broster and Vines describe a fuchsinophil staining reaction exhibited by the cortical adrenal cells in all their cases of virilism. During my recent visit to America I found that this same subject was also interesting their surgeons very greatly, but that they had as yet been unable to reach any generally accepted conclusions. It appears that an identical clinical picture may be given by a cortical adrenal tumour, by adrenal hypertrophy, by a basophil adenoma of the pituitary and by an arrhenoblastoma of the ovary (a tumour probably composed of testicular cells). Further, some patients with typical virilism have died, and at post-mortem no recognisable change has been found in any of their ductless glands. The pathology of this sex change cannot be said to be settled, but in a pronounced case bilateral exploration of the adrenal glands is amply justified.

Two other subjects I will touch upon, because they interest me particularly, but I will do no more since I

discussed them fully in a recent article in the *British Medical Journal.*[1]

Arterial hypertension is a disease that we usually associate with the later years of life. Modern conditions, and particularly the harassing strain and fierce competition of business life in the large cities, are bringing a fresh problem, that of the young man of under thirty who finds himself unable to work because of intolerable headaches, failing vision and inability to concentrate, and who is found to have a systolic blood-pressure of from 200 to 250 mm. of mercury. This type of patient is relatively common in America, and it is in that country that an attempt is being made to provide a surgical treatment for those cases that fail to respond to medical measures. Various operations have been devised, all owing their origin to the observation of the profound drop in blood-pressure that follows the administration of a spinal anæsthetic, and all being designed to produce a similar though permanent dilatation of the vessels in the splanchnic area. Heuer in New York does a laminectomy and divides the anterior roots from the sixth to the twelfth dorsal. Adson of the Mayo Clinic divides the splanchnic nerves below the diaphragm, and also removes the first two lumbar ganglia and part of the semilunar ganglion. Crile removes the whole semilunar ganglion on each side, and divides the branches going to the adrenals. Peet at Ann Arbor removes part of the splanchnic nerves and the lower two ganglia of the thoracic sympathetic chain through a bilateral extra-pleural approach. Of these operations that of Heuer appears to be most severe, that of Peet the simplest and possibly the most satisfactory.

[1] "The Changing Ground of Surgery." *Brit. Med. Jour.*, 4th June, 1938. Vol. i, p. 1,193.

It is very difficult as yet to assess the place, if any, that these operations will ultimately come to occupy in surgery. Two types of case must be excluded at any rate ; those that react well to medical measures and do not need operation, and those that have permanent arterial damage and cannot be improved by any means. When these two groups are eliminated, the number that remains is a very small one, and it appears from a study of the literature of the subject up to date that the majority of the successes of operation are in that small group of young patients without arterial damage who should react to rest, elimination of septic foci, and change of environment. Occasional dramatic successes, such as that of a young surgeon who, having lost his post and abandoned his career because of headaches and impending blindness, was operated on four years ago and has since remained in full work with a normal blood-pressure, serve to emphasize the claims of those who see in the surgery of hypertension the solution for the troubles of civilization.

Ulcerative colitis is not a new problem, but it is a comparatively new one to surgery. The disease appears to be getting commoner, and though we have learned nothing of importance about its cause, we have seen enough of its progress to regard our efforts in treatment hitherto with profound dissatisfaction. Some patients get better after a short course, and remain well. The great majority relapse after a period of improvement, and with succeeding attacks are brought back to a condition of tolerable equilibrium with increasing difficulty, and never emerge from a state of invalidism. The ultimate mortality is close on 100 per cent. The operative treatment is by a complete transverse ileostomy, bringing the whole of the intestinal contents to the surface of the abdomen. The colon, emptied and rested, rapidly becomes sterile. If the ileostomy has been done early in an acute case, the colon may recover its normal appearance and contractability, and it will be possible at a second operation to join up the ileum and restore the alimentary canal to normal. In other cases the large intestine, though recovered from the acute infection, remains scarred and contracted and incapable of resuming its normal function. In such the colon must be resected as soon as the patient is well enough. In a few cases the pelvic colon and rectum recover enough to function again, and the distal ileum may be implanted into the lower colon after the upper portion has been removed. In those with severe ulceration the ileostomy must be looked on as permanent, but the lower ileum soon acquires the power of absorbing water, and the discomfort of the fistula is less than that of a colostomy. The patients, who have perhaps been at death's door for months and only kept alive by transfusions, are restored to robust health and able to resume their normal occupations.

Much more important, to my mind, than any of these recent discoveries of rare diseases or new treatments, is the clarification of our views on some of the more important diseases as the result of new work, and the tendency to an agreed attitude among the majority of experienced surgeons, at any rate, in the English-speaking world.

In peptic ulcer we see a general acceptance, as a working hypothesis at any rate, of the view that gastric and duodenal ulcers are separate and distinct diseases. Gastric ulcer is above all a deficiency disease. Its typical victim is a depressed, overworked and undernourished woman of middle age, with bad teeth or none at all, a long hypotonic stomach whose greater curvature hangs low in the pelvis, and a low acid curve. The ulcer is curable by medical means in its early stages, but if not so cured shows an inclination to chronicity, and when fixed to the posterior abdominal wall becomes entirely resistant. In my opinion it has a definite tendency to malignant transformation. If therefore a gastric ulcer is not cured within a reasonable period by medical treatment, it should be handed over to surgery. The results of partial gastrectomy are excellent, and stand the test of time.

Duodenal ulcer is the outcome of high-pressure living. The typical sufferer is a young business or professional man, married and mortgaged, bolting his meals at irregular intervals between cigarettes and telephone calls. He has a small stomach placed transversely in the epigastrium, rapid emptying, and a high acid curve that ends abruptly at the peak. If the duodenal patient can be persuaded to mend his ways, the ulcer will disappear without surgery; if he cannot, surgery, unless it is of a most drastic nature, will give him only temporary benefit. One of the most manifest changes in the surgical outlook within recent years is that the great body of men of experience and judgement are agreed that in duodenal ulcer operation should be reserved for the complications—perforation, stenosis, severe hæmorrhage—and not be looked upon as a cure for the ulcer tendency.

The treatment of gastric and duodenal hæmorrhage has been the subject of much controversy, but the writers who consider all cases with a history of hæmatemesis together, and do not attempt to separate severe from trivial bleeding, obscure rather than clarify the outlook. Gordon Taylor, Arthur Allen, and Finsterer, on the other hand, have made this distinction, and in consequence their papers suggest a reasoned basis for action. Hæmatemesis in itself, however severe, is no indication for operation ; many of the worst are due to splenic disease, cirrhosis of the liver, or acute mucosal infections. Acute ulcers, again, do not call for surgery. But where a patient with a known chronic ulcer has had a hæmorrhage of a pint or more, operation should be very seriously considered. If there have been previous hæmorrhages of equal severity, or if the patient is over fifty, operation is certainly advisable, and should be undertaken at the safest opportunity.

The decision when to operate in this particular group, the large hæmorrhages from chronic ulcers in patients over fifty, is one of the greatest difficulty, but certain observations will help in this decision. It has been shown that if the bleeding has ceased at the end of twenty-four hours, 60 to 80 per cent. of the patients recover, but if the bleeding still continues up to forty-eight hours 75 per cent. of them die. On the other hand operation in the first forty-eight hours only has a 5 per cent. mortality if it is done by an experienced gastric surgeon under local anæsthesia with a continuous blood transfusion. After forty-eight hours the mortality, even under the same favourable conditions, approaches 80 per cent. Transfusion may bring the hæmoglobin almost to the normal level, but the patient seems to have lost something that cannot be replaced by blood alone, and though it is in the late cases that operation is often most urgently demanded, it is so uniformly fatal that it should not be undertaken. To sum up : when a patient over fifty with a chronic ulcer has a hæmorrhage of over a pint, a physician and a surgeon should examine him together and take joint responsibility for his treatment ; he should be transfused, and the decision whether to operate or not must be made in the first twenty-four hours.

Some of these changes, these crystallizations of opinion, concern what are no more than minor maladies, but are none the less important to patients and therefore to us. I have chosen a group joined by no more secure link than the word "injections," but that word with its vague association with vaccines and hormones, its suggestion of the introduction of mystic fluids "into the system" by routes other than the natural ones, its implied avoidance of "cutting operations" has a great appeal to the public.

The injection of varicose veins has been practised sporadically since the beginning of the century, but it enjoyed a sudden increase in popularity about six years after the war. Various solutions were tried, first salicylates, then quinine and urethane, later sodium morrhuate. Occasional disasters were recorded, and local sloughs were comparatively common at first, but a satisfactory technique was soon elaborated. The outstanding development of the present time is that we have come to realise that the chemical sclerosis of veins can seldom be looked on as a cure, but is rather a temporary palliative or at most an adjuvant to other measures. If the valves guarding the saphenous trunk are incompetent, as they are in practically all patients that come to hospital out-patients complaining of varicose veins, the pressure will always open up a channel, either by dilating collaterals previously invisible, or by canalizing the clot in the main vein. In practice it is found that very few veins treated by injection alone remain obliterated for two years, and many reappear within six months.

The present treatment is by saphenous ligature in all cases that give a positive Trendelenburg test. The old Trendelenburg operation, ligature of the internal saphenous vein in Scarpa's triangle, is insufficient to ensure immunity from recurrence, since blood may reach the trunk lower down by two routes; by anastomoses between the uppermost branches, the superficial external pudendal, superficial epigastric, and superficial circumflex iliac, and the lower ones, the medial and lateral femoral cutaneous; and by communicating veins between the trunk and those under the deep fascia. Now the saphenous vein is exposed two inches below the pubic spine, and all branches between this point and its entry into the femoral vein are divided. The vein is then ligatured above, and divided: and through the lower portion a long needle with a ball point is passed down to the level of the knee, and 5 to 7 c.cm. of sodium morrhuate solution is injected to obliterate all branches and deep communications.

This new Trendelenburg operation can be done at out-patients under local anæsthesia, and gives excellent results. Injection is reserved for purely local varices where Trendelenburg's test is negative, and for sections remaining dilated after ligature.

The injection of hæmorrhoids is a slightly different problem. In varicose veins we inject into a closed space, limited by definite walls with an endothelial lining easily destroyed, and filled with a substance that coagulates and becomes converted into firm fibrous tissue. We destroy the lesion, the whole lesion and nothing but the lesion. Hæmorrhoids are varicose veins, but we do not inject into the veins but round them: and we cure them, not by coagulation, but by producing a submucous fibrosis round them, whose extent is limited only by the amount of the injection and not by any anatomical barrier.

Injection of hæmorrhoids is an excellent palliative when performed by the expert, but it has dangers. Ischio-rectal abscess and fistula may follow and even death from portal pyæmia may occur. An occasional sequel is over-fibrosis, the channel being left rigid and the mucous membrane unduly fixed, so that it cracks and tears with any but the softest motions. Patients like the injection treatment of hæmorrhoids because it avoids the pain of an operation, the danger of an anæsthetic, and the interruptions to work that both imply. Proctologists usually like it, because they acquire the requisite knack in its use. General surgeons see the bad results, and view it with toleration, loathing or fear, according to their experience in this respect. There appears to-day a tendency to limit the use of injection to the treatment of single piles that are bleeding. The relief obtained is never permanent. The modern operation as described by Milligan and Morgan, on the other hand, can be looked on as a permanent cure. A wide V of skin and a smaller one of mucous membrane is dissected inwards off the sphincters as far as the ano-rectal ring at the three primary piles, and the base is ligatured, but no suture is put in. When performed with the cutting electric cautery, combined with Eucupin injection into the rectal wall, this operation is almost painless, and it is perhaps the only one in surgery that gives 100 per cent. good results and no recurrences.

When we come to the injection treatment of hernia, we are departing still further from rational and scientific therapy, for we are injecting not into a vein or round a venous plexus, but into muscular planes. The treatment is intended, not to sclerose the hernial sac, but to produce fibrosis in the inguinal canal by causing a sterile chemical inflammation. The injector therefore sets himself the task of converting the muscles that surround the sac and cord into a sheet of tough fibrous tissue that will contract down on to the sac at its neck sufficiently to obliterate it, yet will not press on the vessels or nerves of the cord. Failure in either direction means that the patient is worse off than before. If the fibrosis is too little, a wide-mouthed sac is reduced to a rigid cartilaginous chink like the canal of a femoral hernia, that will be prone to strangulation ; if it is too much, the cord will be compressed and hydrocele, varicocele, testicular atrophy or neuralgia will follow.

It may seem a waste of time to criticize so futile a method, but I can assure you that the injection of herniæ is a flourishing racket, and you will meet it in Bristol. Various cures of rupture by producing inflammation in the inguinal canal have, of course, been practised from the earliest times by witch doctors and the itinerant quacks who visit fairs, and they occasionally produced cures ; but the method was put on the map by Ignaz Mayer, who introduced the following formula :—

| | |
|---|---|
| Zinc sulphate .. .. .. .. | 1 dr. |
| Phenol crystals .. .. .. .. | 6 dr. |
| Glycerine .. .. .. .. .. | 4 fl. dr. |
| Cinnamon water .. .. .. .. | 1 fl. oz. |
| Fluid extract of Canadian pine (dark) .. | 5 fl. dr. |
| Sterilized chemically purere distilled water | 2 fl. oz. |

Thus, with a convincing patter and a prescription akin to that of the witches in Macbeth, the hernia curer becomes a man of science. If he fails, it can always be said that the Canadian pine was not dark enough or did not really come from Canada. I found in the United States that the injection treatment has been widely tried—for the American surgeon will give anything a trial—but abandoned in all reputable clinics. Their experience is that the pain and suffering involved is greater than with operation and the waste of time much greater ; that at least half of the so-called cures fail ultimately; that of the failures many strangulate ; and that when a patient who has been injected requires operation, either for strangulation or recurrence, nothing can be done to help him, the complete destruction of all layers preventing any satisfactory type of repair.

Discussion of injection leads us to another related subject, the treatment of undescended testes by hormones. It has, in common with the others, the popular appeal of the magic hollow needle, and of operation avoided, but it is a biological and not a local treatment, and has undoubted successes to its credit. Every large hospital has its series of cases treated with Pregnyl or some related substance. That undescended testes descend into the scrotum following hormone therapy has been proved ; but the proportion that do do so is on the whole disappointing, and the symptoms of precocious puberty that usually appear at the same time, especially the enlargement of the penis (often to grotesque proportions) are distressing to the parents, and raise the uncomfortable query whether other endocrine disturbances may not also be taking place.

Observation of patients under treatment over several years, and frank discussion of all aspects of the question at the Association of Surgeons and at medical societies, has led to something like agreement on certain points :—

1. An undescended testicle is not coming to any harm before puberty because of its inguinal position, and is certainly not atrophying.

2. A surprising number of such testes descend at puberty—90 per cent. according to one school doctor.

3. When they do not descend, the cause is nearly always a mechanical block, not a hormonic deficiency.

4. Endocrine injections are called for only when there is evidence of endocrine deficiency—that is, in underdeveloped boys, with excess fat and infantile genitalia. In normally developed children they act only by forestalling what would happen naturally in a year or two. In such normal children they are not merely unnecessary but probably harmful. When one testis is already in the scrotum, to give hormones to help the other is worse than unnecessary, it is half-witted.

5. Operation is unnecessary before puberty unless there is a hernia, or descent is arrested by some obvious mechanical block : after puberty it should always be done.

Talking about recent advances is a lot of fun, and it is in that spirit that we should regard it. The doctor is the guide, counsellor and friend of his patient. He must know something of everything. Above all he must know the broad principles of pathology and treatment that have stood the test of time and stand like rocks eternal. On their surface advances come when the wind blows, and flourish while the sun shines. But they do not greatly alter the outlines of the masses on which they grow.

